# Using electronic tablets for data collection for healthcare service and maternal health assessments in low resource settings: lessons learnt

**DOI:** 10.1186/s12913-019-4161-7

**Published:** 2019-05-27

**Authors:** Fiona M. Dickinson, Mary McCauley, Barbara Madaj, Nynke van den Broek

**Affiliations:** Centre for Maternal and Newborn Health, Liverpool School of Tropical Medicine, Pembroke Place, Liverpool, L3 5QA UK

**Keywords:** Electronic data collection, Health research, Lessons learned, Low- and middle-income countries

## Abstract

**Background:**

Health service and health outcome data collection across many low- and middle-income countries (LMICs) is, to date largely paper-based. With the development and increased availability of reliable technology, electronic tablets could be used for electronic data collection in such settings. This paper describes our experiences with implementing electronic data collection methods, using electronic tablets, across different settings in four LMICs.

**Methods:**

Within our research centre, the use of electronic data collection using electronic tablets was piloted during a healthcare facility assessment study in Ghana. After further development, we then used electronic data collection in a multi-country, cross-sectional study to measure ill-health in women during and after pregnancy, in India, Kenya and Pakistan. All data was transferred electronically to a central research team in the UK where it was processed, cleaned, analysed and stored.

**Results:**

The healthcare facility assessment study in Ghana demonstrated the feasibility and acceptability to healthcare providers of using electronic tablets to collect data from seven healthcare facilities. In the maternal morbidity study, electronic data collection proved to be an effective way for healthcare providers to document over 400 maternal health variables, in 8530 women during and after pregnancy in India, Kenya and Pakistan.

**Conclusions:**

Electronic data collection provides an effective platform which can be used successfully to collect data from healthcare facility registers and from patients during health consultations; and to transfer large quantities of data. To ensure successful electronic data collection and transfer between settings, we recommend that close attention is paid to study design, data collection, tool design, local internet access and device security.

**Electronic supplementary material:**

The online version of this article (10.1186/s12913-019-4161-7) contains supplementary material, which is available to authorized users.

## Background

To date, most data collection in health research in low resource settings has used paper-based data collection tools. Globally, there has been an increase in the availability, quality and affordability of electronic mobile technology such as telephones, personal digital assistants and electronic tablets. In high-income countries (HIC), computerised technology and mobile electronic devices are part of daily life with 83–90% of people aged 16–54 years in the UK owning a smartphone [1]. Mobile phone subscription has increased globally from 12% in 2000 to 97% in 2014, in sub-Saharan Africa it increased from 2 to 70%, and in India from 0 to 73% [2]. Alongside an increase in the development and use of computer technology, electronic data collection is increasingly being used for healthcare implementation and research in HIC [3–6]. Whilst there are reports of electronic data collection from low-income countries using mobile phones and personal digital assistants (PDA) [3, 7–9], there is less information regarding the feasibility, acceptability and practical challenges with the use of electronic tablets for data collection, from these settings [10, 11].

Electronic data collection has several advantages compared to paper-based collection, including enabling large volumes of data to be collected and stored securely by means of password protection and data encryption on an electronic tablet, and avoiding the need to carry and store bulky paperwork. Electronic data can be transmitted securely and quickly, using existing 3G mobile phone networks or Wi-Fi connection to a remote research base, allowing data to be processed, reviewed and disseminated quickly. Another advantage of electronic data collection is the ability to impose validation rules as data is entered (reducing the risk of human error) and the decrease in time necessary for data entry and cleaning following the data collection phase of a study [7, 12, 13].

There are many different software packages available for collecting electronic data. These include basic ‘one question at a time’ applications designed to be used on mobile phones, to more complex packages with multi-stage, multi-user validations that can be used on laptops or desk-based computers.

The Centre for Maternal and Newborn Health (CMNH) at the Liverpool School of Tropical Medicine (LSTM), has supported the collection of health research data using paper-based questionnaires and surveys on a monthly or quarterly basis in more than 1000 healthcare facilities across 11 countries since 2006. With progress in the availability and quality of electronic devices in low resource settings, alternative options for a more efficient method of data collection were explored, primarily focusing on electronic tablet-based data collection.

This paper describes our experiences and lessons learnt with regard to implementing electronic-tablet-based data collection methods for healthcare facility assessment and to collect maternal health data from women during and after pregnancy across different settings in four LMICs. We set out what worked where and how and provider recommendations for the selection and use of electronic data collection tools and practice. In particular, we explore the use of paper versus electronic tools and comment on a variety of software packages including Filemaker and Excel.

## Methods

### Choosing electronic data collection tools

To identify a suitable electronic data collection software package, we identified specific requirements, based on our previous experience of collecting data in LMICs (Table 1). Key requirements included (1) the ability to collect data without an internet connection (but with the ability to transmit data to the UK when necessary using Wi-Fi or cell phone networks) (2) the ability to work well with small (50–100) as well as large-scale (> 100) sample sizes (patients, healthcare facilities) and (3) ensuring an in principle low-cost application and use. An initial literature and internet search was carried out to identify existing suitable software packages, with no restriction of platform (e.g. android or Apple-iOS). In addition, mHealth Knowledge an external organisation specialising in connecting global health professionals with electronic resources, was consulted [14].

**Table 1:** Essential requirements for electronic data collection

	Essential requirements
Internet	• Usable off-line
	• Data can be stored securely until checked for completeness
	• Able to be uploaded to server
Platform	• Can be used on electronic tablets to facilitate viewing multiple questions at same time
Question types	• Tick box
	• Number box
	• Short free text
	• Date
	• Likert scale
	• GPS location capture
Design features	• Skip logic & answer validation
	• Ease of design – Graphical user interface
	• Potential to create sub-forms within a tool
	• Ease of deployment
System features	Able to facilitate:
	• Large number of uploads (> 15,000/annum)
	• > 500 questions /form
Data analysis	• Data exportable to Excel/SPSS
Support	• Support for staff
	• Training
Cost	• Not significantly more than existing paper-based option

### Comparing EDC with excel® and paper-based tools

Filemaker® was used alongside a paper-based software package (Formic®), as part of an ongoing, routine healthcare facility assessment in Ghana (the Making it Happen programme). Data collection was conducted in the Western Region in seven healthcare facilities. The electronic data collection using Filemaker® software was deployed on different sizes of iPad (cellular mini and standard) to ascertain data collector’s preferences for portability versus increased screen size. For comparison purposes, the same data collection tool was also developed on Microsoft Excel and used on a laptop alongside the tablet-based and paper-based methods.

Prior to the start of the data collection, a half day training session was provided for the five data collectors, all of whom were already familiar with the paper-based tool and four of whom had personal smart phones. Data collectors then worked in two groups to collect data in healthcare facilities from facility registers, as part of an evaluation of a maternal health intervention program. Simultaneous data collection using Filemaker® and paper forms was carried out, with data collectors alternating between the different methods at different facilities. As it would have been too difficult to collect data ‘live’ using the Excel form as well, this was carried out as a separate activity at the end of the day. All uploading and processing of data was conducted after data collection was completed, in the central office.

In order to assess the ease of use of the tools by the data collectors, a feedback form was developed (Additional file 1). This asked about the time taken to complete the tools using each method, ease of use, and any problems encountered, as well as allowing space for other comments.

### Maternal morbidity study

Subsequently, electronic data collection was then used as part of a separate multi-country, maternal health survey to measure the burden of ill-health in women during and after pregnancy in three LMIC (India, Kenya, and Pakistan) across 20 healthcare facilities (India: 1, Kenya: 9, Pakistan: 10). Trained healthcare providers conducted all activities and electronic tablets were used to input all data [15].

Mini sized cellular iPads were used for electronic data collection. To reduce the risk of damage, inexpensive covers and screen protectors were used. Additional electronic tablets were allocated to the research supervisors in each country to ensure that data collection could continue even if there was a problem with one of the electronic tablets.

#### Training

As part of the five-day study specific training, half a day was specifically allocated to the use of the electronic tablets, as well as the use the forms and upload and transfer of completed data. Data collectors were also briefed on appropriate methods of cleaning, transporting and storing the electronic tablets to reduce the likelihood of damage occurring. Written instructions were also provided and contact details for research supervisors in each country were made available to help with any technical issues.

Electronic data collection using Filemaker® software was used as the primary means of data collection for the maternal morbidity study. A total of 32 data collectors used 49 electronic tablets to collect data across three countries (India: 5, Pakistan: 19, and Kenya: 25).

All data collectors were trained healthcare providers (nurses, midwives, doctors), working in the study healthcare facilities, with varying levels of experience of using electronic devices.

A total of 8531 women were assessed (India: 2099, Kenya: 3145, and Pakistan: 3287). Data was collected from women at different stages of pregnancy; at primary and secondary level healthcare facilities; in different settings (urban and rural) [15].

#### Electronic tool development

The main requirement of the use of electronic data collection for the maternal morbidity study was to be able to upload data from the electronic tablet directly to an externally hosted server in the UK, a process that required specific scripting. The tool consisted of approximately 400 variables with in-built validation and skip logic where questions and answers permitted. An external Filemaker® consultant developed the tool for the data collection in India. However, due to lack of availability of the same consultant, it was not possible to use this approach in Kenya and Pakistan. Therefore, data collectors were not able to upload data directly to the UK based server, and this was resolved by emailing data from the devices to a dedicated email address, which were then downloaded and transferred onto the database by a researcher in CMNH. The form was loaded onto the electronic tablets by a member of the research team as part of the study data collector training in India. The Pakistan and Kenya versions of the tool were uploaded onto the electronic tablets prior to use in the relevant countries. In all three cases the forms were loaded onto the tablets using a single country specific webmail account previously added to each device. This enabled the form to be loaded onto multiple electronic tablets from a single email. As an additional measure to ensure no loss of data occurred, all data was stored on the electronic tablets until they were returned to the CMNH, where the data was cross checked with that on the merged database and then deleted.

#### Security

To protect data collected but also minimise the risk of misuse by third parties, each electronic tablet had a passcode enabled, so that the device could not be used without entering the correct four-digit code. Additionally, all devices were ‘restricted’ prior to use by means of disabling all the non-study specific applications on the device. This meant that data collectors could not use the device for personal emails, accessing social media, the internet, or any other personal activity. Data collectors were given the access code to their own device, but only the national co-ordinator had the ‘master list’ of access and restriction codes. Additional in-built security features on the devices included the ability to remotely locate and track them when switched on and if stolen to remotely lock them and if necessary wipe all data from the tablet. This was a particularly important feature considering the nature of the data on each device, even though the data were anonymous and non-identifiable in accordance with the Data Protection Act and ethical procedures.

## Results

### Choice of software package

The process of comparing identified software packages with our data collection requirements resulted in several potential options (Table 2) which were each considered individually. A number of potential software packages were excluded on the basis of either not being available ‘off-line’, being too simple only showing one question at a time or being too complex and correspondingly more expensive, at time of review.

**Table 2:** Electronic software identified and considered for use

Software	Developer	Reason for non-selection
Access	Microsoft	Not available on electronic tablets
BOS (now Online surveys)	JISC	Not available off-line
Clincapture	Clinovo	Cost
Clinplus	Anju ClinPlus	Cost
Excel	Microsoft	NA
Filemaker Pro	Filemaker	NA
KoBo toolbox	Harvard Humanitarian Initiative	Problems with versions of Java, only accessible using Firefox browser
Magpi	Magpi	Cost, lack of necessary data validation options
ODK	ODK Community	Form design - lack of graphical user interface
ONA	Ona Systems	Form design - lack of graphical user interface
Quick tap survey	Quick Tap Survey	Form design
Survey system	Creative Research Systems	Cost

After consideration of these options, in our case, Filemaker®, an Apple subsidiary was chosen. For our purposes, this was considered the most suitable because of: costs, ability to collect data off-line, whilst still being able to allow complex internal validation and skip patterns.

Filemaker® had three main formats: “Pro” – for form design and data handling (available for Apple and Microsoft computers); “Server” – for data storage; and “Go” (an app) – for data collection on electronic tablet. The Pro version used ‘drag and drop’ methods to allow new users to design simple, useable solutions quickly, whilst more competent specialists could use scripting to develop more complex relational databases and data collection tools [16].

### Comparison of paper-based and electronic formats

In total, data was successfully collected from seven hospitals and health centres, using electronic tablets and paper-based forms in all instances. Qualitative feedback from all the data collectors confirmed Filemaker® as their preferred option, with no significant problems reported that compromised data collection by either impeding or impairing the process. Most data collectors preferred the mini electronic tablet due to its lighter weight and easier portability. However, one data collector with sight problems, expressed a preference for the larger, standard sized device, as it was easier to see the text. It was possible to resize the text using ‘two finger pinch’ methods, however as this was her first time using touch screen devices she lacked skill and confidence in this technique. Data collectors felt that the extra encumbrance of carrying and using the laptop in often cramped and busy clinical work areas outweighed any benefits from familiarity with the Excel version. No significant difference was noticed between the two sets of data produced, although the paper forms required extra processing time after data collection, to convert into electronic format and clean the data prior to analysis.

The advantages and disadvantages of the electronic data collection methods are summarised in Table 3).

**Table 3:** Summary of the advantages and disadvantages experienced using electronic data collection

	Advantages	Disadvantages
Software	• Automated validation and skip logic capacity	• Time required to familiarise with new software
	• Off-line data collection capacity	• Specialist technical help required to develop script for uploading data to server in the UK
		• Training required to design and manage forms
Hardware	• Portability of electronic tablets Option to resize screen to enlarge text useful for data collection	• Issues with shipping devices and customs clearance for international deployment
		• Increased risk of theft of tablets compared to paper forms
Costs	• Re-usability of electronic tablets following initial investment	• Cost of electronic tablets and accessories (covers, chargers, etc)
		• Cost of software
		• Cost of external support for technical aspects (scripting)
Field -based Training	• Data collectors enjoyed training and appreciated capacity building	• Training required to use devices
		• Training required to use software for data collection
Security	• Data password protection on devices	
Processing and cleaning	• Reduced need for processing and cleaning of data compared to paper forms	• Time required for inbuilding validations and skip patterns in the tool at development stage

### Use of tool in the maternal morbidity study

#### Processing and cleaning

Data from India was uploaded directly to the server and therefore required minimal processing to prepare it for analysis. It was downloaded from the Filemaker® server directly to Microsoft Excel for cleaning and merging with the other data, to form a single overall study dataset. Data from Pakistan and Kenya was sent using email and required more processing to extract the data from the emails and compile into a dataset. Specifically, processing and cleaning included ensuring data was received, entered into appropriate template and assessed for quality (missing data, wrong data) and where necessary corrected to reflect the actual state.

#### Hardware

Electronic tablets were purchased in the UK to allow the tablets to have the necessary software added and restrictions enabled, then shipped using international courier services. This did entail some delays and issues with obtaining customs clearance. No significant problems were experienced with using the electronic tablets and all the devices were returned undamaged and in good working order.

#### Costs

For both studies, the cost of tablets was similar; each device cost approximately £270 (March 2015) to purchase including: cost for the institution name and a unique ID number to be engraved on each device, a book style cover and screen protector. A total of six Filemaker Pro® and one Filemaker Pro Advanced® licences were purchased on an annual renewal arrangement, costing approximately £480 annually. This allowed flexibility for increasing or decreasing the number of licences as software needs changed and providing periodic updates at no extra cost.

## Discussion

To the best of our knowledge, this is the first paper describing the experience of electronic data collection using electronic tablets, across four countries, at patient and healthcare facility level. We highlight the advantages as well as potential issues in using electronic tablets for health research, sharing experiences and lessons learned using Filemaker® as a means of developing data collection tools on electronic tablets as a platform for electronic data collection. Although the tools used were complex, no significant problems were experienced with data collection. Issues such as the availability of electricity supply and internet connection, security of potentially valuable equipment, as well as the need for additional training prior to the start of data collection, may well be encountered across different electronic data collection platforms.

Overall, electronic data was successfully collected in and transferred from all settings using electronic techniques. Both studies illustrate some of the advantages and disadvantages encountered when using electronic tablets for collecting health data in LMIC settings. It was demonstrated that it is possible to use modern electronic methods to carry out data collection in LMIC countries, using healthcare providers as data collectors in their relevant workplace. In the maternal morbidity study, electronic data collection was a feasible and effective method of data collection, to document over 400 variables for 8530 women.

Although the cost of the electronic tablets was a significant outlay at the beginning of the study (£1350 for the five devices deployed), these were subsequently reused making them an investment for organisations carrying out multiple research or similar projects. Calculating a meaningful cost analysis for the possible data collection methods is difficult, however, if the data collection for the maternal morbidity study had been carried out using paper forms, the printing cost alone would have been approximately £5000. In addition to this it would have been necessary to increase the staff time spent processing the forms.

### Hardware and security

Initially, there were concerns regarding the risk of electronic tablets being lost, damaged or stolen, however in these studies concerns were proven to be unfounded. Other researchers reported having to replace 11 broken chargers, two batteries, three stolen SD (Secure Digital) cards and four electronic devices (from a total of 64) which they considered as not unreasonable [8]. They also report having a total of six out of 64 data collection devices stolen in another study. This may in part have been due to the use of commercial charging facilities and using relatively small ‘pocket sized’ devices such as smart phones and personal digital assistants. We intentionally invested in a relatively larger size of the electronic tablets and brightly coloured covers. During training, data collectors were strongly encouraged to place the devices securely and out of sight in a bag when not in use. The collection of data in a relatively secure setting such as a healthcare facility might also have been beneficial in relation to safety of the devices. The use of wrap-around, ‘book style’ covers, although inexpensive almost certainly prevented damage to the electronic tablets. A few damaged covers were replaced following the Kenya phase of the study, though this may have been due to damage in transit rather than in use and the occasional cost of a replacement cover (approx. £5) compared to the cost of an electronic tablet (£250–300) offers relatively good value.

There are, however, some potential challenges to using electronic data collection, primarily related to the fact that the devices need to be charged regularly. In one study in Burkina Faso, researchers used a combination of mains charging where available, charging from vehicles using a cigarette lighter adapter, 12-V batteries and solar panels to resolve problems with irregular electricity supplies [9], and other researchers used “commercial charging services” in one study in Malawi [8]. In our studies, the tablets were charged overnight by the data collectors without problem, either at home or in a hotel. However, subsequently we have also used spare battery packs and cigarette-lighter adaptors for use when travelling in a suitable vehicle to avoid any problems with power cuts.

### Data transfer and processing

Considering the risk of loss of electronic data collection devices and the consequential loss of data, communicating the data to a secure server was particularly important. We could do this effectively using Wi-Fi and (3G sim cards), although in areas and countries with intermittent network coverage this may not be possible immediately. The increased time needed to download and process the emailed data from Pakistan and Kenya was unforeseen and unfortunate, however in our experience it was less than that required to process the paper forms, either using optical character recognition processing software or manual data entry, particularly with their inherent risk of human (or machine) error in reading and or entering the data. The requirements for scanning and processing paper forms varies considerably depending on the length of the form and the quality of the data entry but on average it took approximately 45 min to scan and process a 14-page healthcare facility assessment form, whilst downloading a similar form electronically took less than 5 min.

### Limitations of the study

The two studies detailed were carried out in the relatively secure setting of a healthcare facility. Carrying out data collection in a household or other community setting might bring additional challenges, particularly relating to security, electricity supply and internet connectivity.

The comparison of methods of data collection was done using a pragmatic approach rather than a specific framework and therefore there is no comparison of the two sets of data collected during the Healthcare Facility Assessment. However, data generated by the two methods did not yield any specific differences regarding the content of the datasets, only the process of data collection and processing as outlined above.

A future study comparing the current state of the art technology may well be beneficial, but it will be important to consider the cost and complexity of comparing multiple platforms, and the speed with which technology advances. Mobile technology and data collection software is constantly evolving and improving, with new products continually coming on to the market. Since this study, other products will already have become available, which might be cheaper and arguably better suited to health data collection, particularly using android-based devices and automated data uploads.

## Conclusion

This paper aimed to set out lessons learnt when moving form paper to electronic data collection methods and tools. Electronic devices can be successfully used in low- and middle-income settings and data collectors generally like them. Data collection and processing are much easier but there can be problems with connectivity and with availability of electricity to recharge devices. Technology is continuously evolving and when devices need to be made to change methods and tools of data collection these are factors that merit consideration.

## Additional file

Additional file 1: EDC pilot feedback form. (DOCX 22 kb)

## Abbreviations

CMNH: Centre for Maternal and Newborn Health
HIC: High-Income Country
LMIC: Low- and Middle-Income Country
LSTM: Liverpool School of Tropical Medicine
PDA: Personal Digital Assistant
SD card: Secure Digital card
