# Supplementary material for: Using electronic tablets for data collection for healthcare service and maternal health assessments in low resource settings: lessons learnt
Source: BMC Health Serv Res. 2019 May 27;19:336. doi: 10.1186/s12913-019-4161-7 (PMC6537391; doi:10.1186/s12913-019-4161-7)
Supplement: Supplementary file 1 — EDC pilot feedback form. (DOCX 22 kb) [file 12913_2019_4161_MOESM1_ESM.docx]

**EDC pilot feedback form**

1. Which data collection tools did you use?

- Paper
- Filemaker
- Excel

1. Approximately how long did it take to complete the electronic forms in relation to the paper forms?

- Filemaker ____________________________________________________________
- Excel ________________________________________________________________

1. How easy did you find them to use?

- Filemaker__________________________________________________________________
- Excel ______________________________________________________________________

1. Did you have any problems using the electronic forms?

- Filemaker___________________________________________________________________
- Excel _______________________________________________________________________

1. How easy did you find the electronic tablets to use?

- Filemaker __________________________________________________________________
- Excel ______________________________________________________________________

1. How did you find uploading the information after collection?

- Filemaker __________________________________________________________________
- Excel ______________________________________________________________________

1. Any other comments?
